# Appetitive floral odours prevent aggression in honeybees

**DOI:** 10.1038/ncomms10247

**Published:** 2015-12-22

**Authors:** Morgane Nouvian, Lucie Hotier, Charles Claudianos, Martin Giurfa, Judith Reinhard

**Affiliations:** 1 Queensland Brain Institute, University of Queensland, Brisbane, Queensland 4072, Australia; 2 Centre National de la Recherche Scientifique (CNRS), Centre de Recherches sur la Cognition Animale (UMR5169), 118 route de Narbonne, 31062 Toulouse, Cedex 09, France; 3 UPS Centre de Recherches sur la Cognition Animale (UMR5169), Université de Toulouse, 118 route de Narbonne, 31062 Toulouse, Cedex 09, France; 4 School of Psychological Sciences, Monash University, Melbourne, Victoria 3600, Australia

## Abstract

Honeybees defend their colonies aggressively against intruders and release a potent alarm pheromone to recruit nestmates into defensive tasks. The effect of floral odours on this behaviour has never been studied, despite the relevance of these olfactory cues for the biology of bees. Here we use a novel assay to investigate social and olfactory cues that drive defensive behaviour in bees. We show that social interactions are necessary to reveal the recruiting function of the alarm pheromone and that specific floral odours—linalool and 2-phenylethanol—have the surprising capacity to block recruitment by the alarm pheromone. This effect is not due to an olfactory masking of the pheromone by the floral odours, but correlates with their appetitive value. In addition to their potential applications, these findings provide new insights about how honeybees make the decision to engage into defence and how conflicting information affects this process.

## Abstract (web summary)

Honeybee olfactory systems may experience conflicting cues from communication- and foraging-related odours. Here, Nouvian *et al.* find that appetitive signals from floral compounds can reduce aggression by blocking the recruitment to defence triggered by alarm pheromones, and that this is not due to odourant masking.

## Graphical abstract

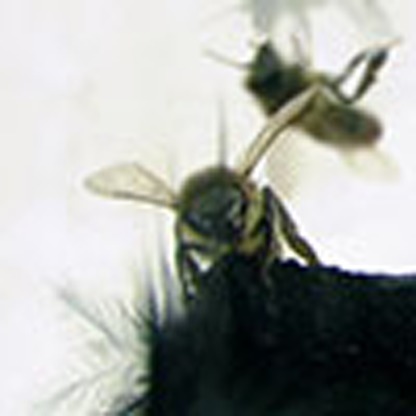

Aggression is a crucial element in the competition for food, mates and territory, as well as a defence mechanism against predators. The defensive behaviour of the honeybee *Apis mellifera* aims at the protection of its nest, which contains the food, the brood and the only reproductive individual of the colony, the queen. A specific subset of worker bees, the guards, are responsible for responding to any disturbance occurring close to the colony^1^. Guards are highly responsive to visual cues such as movement and dark colours, which allow them to identify and locate potential intruders^2^. Guard bees signal this threat to soldier bees^3^ inside the nest by releasing the sting alarm pheromone (SAP)^4^, which triggers collective aggressive responses. Over 40 compounds have been identified in this pheromonal blend but its main component, isoamyl acetate (IAA), is sufficient to elicit most of the behavioural response to SAP^5^,^6^. When bees are stimulated by SAP, excitement soon unfolds and they fly out, harass and eventually sting the intruder^7^; if the stinger apparatus pierces elastic tissue such as human skin, it is detached from the abdomen and stays in the wound after stinging, causing the death of the mutilated bee a few hours later^8^,^9^,^10^. This extreme cost of aggression may explain why engaging into defensive behaviour is tightly regulated both at the individual and colony level by a variety of factors such as the foraging conditions^11^, the state of the reserves of the colony^12^ and the defensiveness of a guards' nestmates^13^,^14^,^15^,^16^, to name a few. However, the mechanisms underlying this regulation remain to be elucidated.

Olfaction plays a major role for worker honeybees in a variety of behavioural contexts including nest defence^17^. Many odourants (in particular pheromones) are only released in a specific context and thus trigger stereotyped behavioural responses^18^. Yet, odour-specific behaviours may be subject to the modulatory action of odourants that are ostensibly irrelevant for the task considered. For instance, exposure to the SAP impairs appetitive olfactory learning in which bees learn to associate a neutral odourant with sucrose solution^19^. In this case, the learning impairment may be a response to the alarm signal, which would detract bees from responding to appetitive stimuli in a situation in which such responses would be of secondary importance compared with hive defence. In this study, we investigated the reverse scenario, that is, whether plant odours, referring to an appetitive foraging context, affect the response to the alarm pheromone in a defensive context. Semiochemical interactions between two sets of odours are known to occur in insects: in the moths, for example, host plant odours affect the response to sex pheromones and *vice versa*^20^,^21^,^22^,^23^,^24^.

The aggressive behaviour of the honeybee is an excellent model to study this question, because it is reliably triggered by a pheromonal odour (IAA) and results in a stereotypic and easily measured behaviour (stinging). Here we demonstrate for the first time that the floral compounds linalool (Lol) and 2-phenylethanol (PhE), as well as the odour mixture lavender (Lav), block the aggressive response triggered by IAA. This decreased response is not due to IAA being masked by these specific compounds, but rather correlates with the fact that these floral odours act as appetitive signals for bees. The fact that honeybees weigh and integrate different olfactory stimuli before taking action provides new insights regarding the possible neural circuitry that regulates aggressive behaviour. Furthermore, determining whether and how honeybee aggression can be modulated by exposure to natural odourants that are not related to a defensive context may have important practical and economic implications.

## Results

### Effect of IAA and social interactions on honeybee aggression

**Figure 1:**
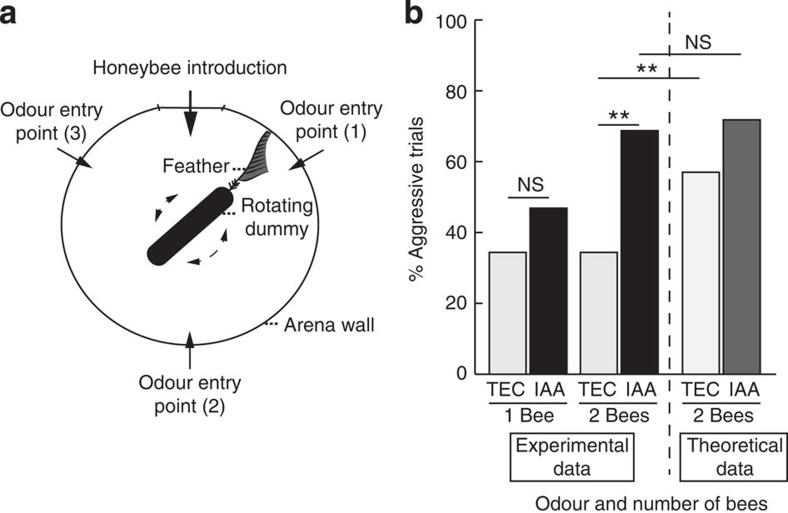
Aggression assay. (**a**) Top view of the arena showing the location of the odourant and honeybee entry points, as well as the rotating dummy. (**b**) ‘Experimental data': percentage of trials in which at least one of the bee stung the dummy, recorded as a function of the odour present (TEC: solvent; IAA: alarm pheromone) and the number of bees introduced inside the arena. ‘Theoretical data': results expected if the two bees were acting independently from each other, calculated from the probability of attack of a single bee. *χ*^2^ tests; NS, not significant; *P*>0.05, **P<0.01, *n*=32 single bees and 32 pairs of bees.

To investigate honeybee aggression in a controlled environment, we developed a novel assay in which individual or small groups of bees are confronted with a moving, dark target (a rotating dummy) inside a cylindrical arena (14 × 4 cm) into which various odours can be released via an automated olfactometer (Fig. 1a). Honeybees involved in colony defence (guards and soldiers) were selected from natural hives by briefly waving a black feather in front of the colony entrance and collecting the bees attacking the feather. After a short cold anaesthesia and at least 15 min of recuperation, the bees were tested for their aggressive behaviour towards the target in the arena for 3 min in the presence of different odours. Aggressiveness was measured as the percentage of trials during which at least one bee attempted to sting the dummy.

As it was previously reported that single workers rarely respond to SAP, and that the defensive behaviour of honeybees is subject to a positive group effect^15^, we first evaluated the aggressive response of single (*n*=32 bees) or paired honeybees (*n*=32 pairs). The bees in the arena were exposed either to a solvent control (triethyl citrate, TEC) or to IAA (10% in TEC, Table 1). When a single bee was inside the arena, no significant difference between the proportion of attacks to the dummy was observed between IAA- and TEC-exposed bees (*χ*^2^=1.036, df=1, *P*=0.309), although a slight increase in aggressiveness could be observed in the presence of IAA (Fig. 1b). When pairs of bees were tested in the arena, they reacted strongly to IAA and increased significantly their attacks compared with the controls (69 versus 34%, respectively; *χ*^2^=7.570, df=1, *P*=0.006).

Although these experimental results are in agreement with the previous observations of a positive group effect during honeybee aggression^15^, they do not exclude a purely additive rather than a synergistic effect. To decide between these alternatives, we calculated the hypothetical aggression levels of paired bees under the assumption that social interactions would have no effect on aggression, that is, the bees in the arena would behave as single bees. In this scenario, the aggressive behaviour recorded for the bee pairs would be purely additive. The results of this calculation are presented in Fig. 1b as ‘Theoretical data'. Surprisingly, comparison of these theoretical values (paired bees acting independently) with the experimental data (paired bees acting socially) revealed that social interactions did not cause an increased response to IAA (*χ*^2^=0.148, df=1, *P*=0.700). Rather, social interactions between paired bees led to a decreased baseline of aggressiveness in the absence of IAA, as the proportion of aggressive trials in the TEC-exposed group was lower in the experimental group than in the theoretical group (*χ*^2^=6.683, df=1, *P*=0.010). Although the reasons for this lowered baseline of aggression in paired bees remain to be determined, this experiment highlights that the presence of another bee in the arena is necessary to reveal the natural recruiting function of IAA in our experimental set-up. Therefore, we used pairs of bees to investigate the role of olfactory cues on aggressive behaviour.

### IAA-induced aggression is blocked by specific floral odours

Next, we studied the effect of several plant-derived odours and one other pheromonal compound (citral; Ci) on the aggressiveness of honeybees (see Table 1). We exposed the bees to these odourants, either on their own or in combination with IAA, while confronting them with the dummy in the arena. The test odourants were chosen among the most common floral compounds, which are likely to be encountered by bees. Praescent (Pr), a mixture of plant-derived odours, was also chosen because of its known relieving effect on vertebrate stress^25^,^26^. Indeed, honeybee colonies are known to become more aggressive when the resources are scarce^11^ (that is, in response to a stressor); hence, we wanted to explore whether Pr could also modulate honeybee aggression. IAA was presented as a 10% (vol/vol) solution, whereas all other odourants were at 0.075% (Table 1). This ratio was chosen to ensure the salience of IAA. As it was not technically possible to test all the odourants simultaneously, they were divided into four sets of experiments. TEC and IAA stimulations were included in all four sets and served as references. Values obtained for each of these two reference stimulations did not differ across the four set of experiments (TEC and IAA, generalized linear model (GLM), *P*>0.05 in both cases); hence, the data were pooled. As a result, the data points for TEC and IAA include 128 pairs of bees, whereas all the others include 32 pairs.

**Figure 2:**
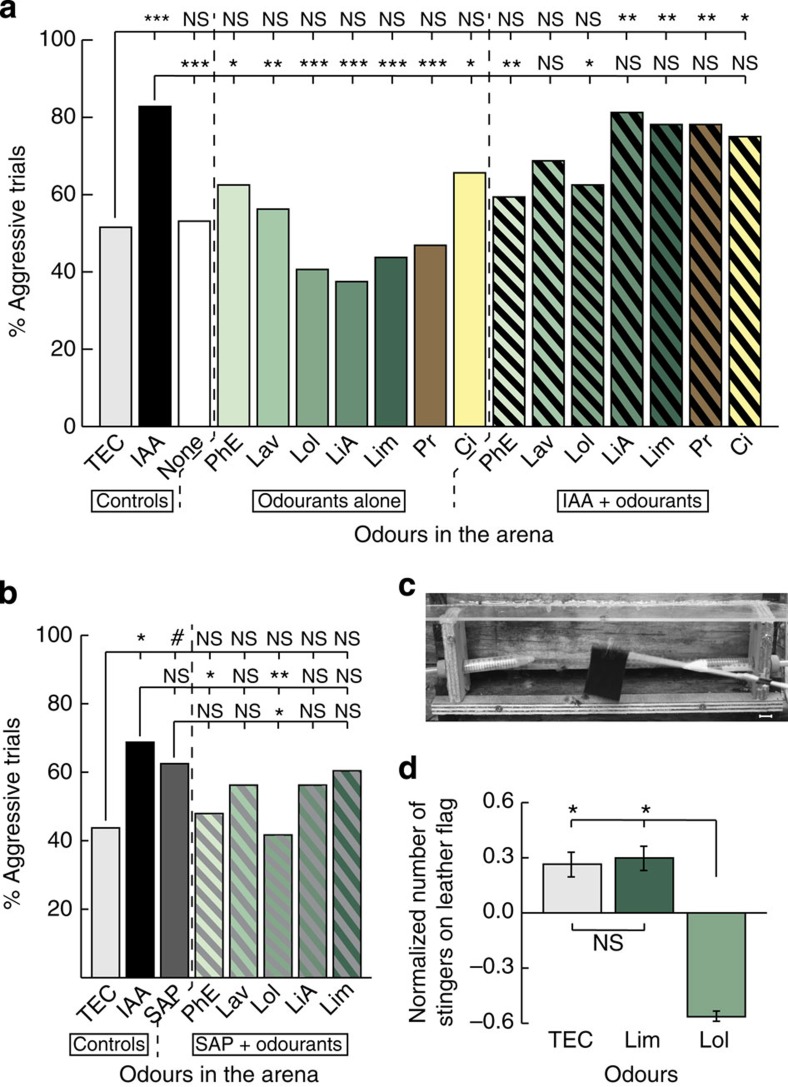
Some floral odours block the aggressive response to the alarm pheromone. (**a**) Percentage of aggressive trials recorded as a function of the odours blown inside the arena. ‘Controls' include TEC (solvent), IAA (alarm pheromone) and None (no odour). ‘Odourants alone' shows that when the compounds were not associated with IAA, none of them had an impact on aggression. In the ‘IAA+Odourants' section of the graph, the same compounds are presented alongside the alarm pheromone. PhE and Lol significantly decrease the response to IAA and Lavender to a lesser extent. GLM; NS, not significant; *P*>0.1, *P<0.05, **P<0.01, ***P<0.001, *n*=128 pairs of bees in the TEC and IAA groups, and *n*=32 pairs in all the other groups. (**b**) Percentage of aggressive trials recorded as a function of the odours blown inside the arena. ‘Controls' include TEC (solvent), IAA (main component of the alarm pheromone) and SAP (sting alarm pheromone). The floral compounds have similar effects when presented alongside SAP (‘SAP+Odourants') than when they were presented alongside IAA. GLM; NS, not significant; *P*>0.1, #*P*=0.067, *P<0.05, **P<0.01, *n*=48 pairs of bees in each group. (**c**) Field test set-up around the entrance slit of a hive. The landing board, small wooden walls and plastic roof form the sides of the box (open here) used to create a stable atmosphere for odour delivery, which is done through the Falcon tubes. The black leather flag is jiggled via a small motor (not visible on the picture). Two aroused bees can be seen under the flag. Scale bar, 1.5 cm. (**d**) Number of stingers embedded on the leather flag depending on the odours blown in front of the hive, normalized per colony (mean±s.e.m.). Lol significantly decreases the number of bees engaging into defence of the colony. ANOVA with repeated measures, Bonferroni-corrected threshold *α*=0.017; NS, not significant; *P*>*α*, *P<*α*, *n*=16 trials per odour treatment.

As expected (see ‘Controls' section of Fig. 2a), the bees attacked the dummy much more frequently when IAA was blown inside the arena than when there was only TEC, the solvent control (GLM, *P*<0.001), thus confirming the data from the first experiment (Fig. 1b). We also included a control with no odour (None) that resulted in a level of aggression similar to the one occurring with TEC, confirming that the solvent itself did not have any effect (GLM, *P*=0.874 versus TEC, *P*<0.001 versus IAA).

When the bees were exposed to plant odours alone (Fig. 2a ‘Odourants alone', green and brown bars), their aggressiveness did not differ from the baseline level measured during the TEC trials (GLM, all *P*-values >0.1). In all cases, aggression levels remained significantly lower than that displayed during IAA trials (GLM, *P*<0.05 to *P*<0.001 for all comparisons). The pheromonal compound Ci, which is part of the attractant ‘Nasonov' pheromone^27^, did not have any effect either (GLM, *P*=0.156 versus TEC and *P*=0.035 versus IAA). However, when the same odours were presented simultaneously with IAA (Fig. 2a, IAA+Odourants), different types of response could be observed. On the one hand, addition of linalyl acetate (LiA), limonene (Lim), Ci or the odour mixture Pr did not affect the response to IAA. When bees were exposed to these odourants combined with IAA, the percentage of aggressive responses measured was significantly higher than the baseline control (GLM, *P*<0.05 to *P*<0.01 versus TEC for all comparisons) and similar to the aggression level elicited by IAA alone (GLM, *P*>0.1 for all comparisons). By contrast, bees exposed simultaneously to IAA and PhE, or IAA and Lol, did not attack the dummy as frequently as those exposed to IAA alone (GLM, *P*<0.05 versus IAA in both cases). In these cases, the bees' aggressiveness was reduced to levels similar to the baseline (GLM, *P*>0.1 versus TEC in both cases). Finally, the mixture Lav, which is composed of Lol and LiA (Table 1), presented together with IAA provoked an intermediate state where the percentage of aggressive trials was not significantly different from that induced by IAA alone (GLM, *P*=0.084) but also similar to the one of the solvent control (GLM, *P*=0.080). As this mixture is composed of Lol and LiA, the small reduction of aggression may be driven by the presence of Lol.

Although IAA is sufficient to trigger a full defensive response, over 40 compounds have been identified in the honeybee SAP^6^,^28^. We therefore also investigated the effect of natural SAP on aggressiveness. We excised 30 stings from defensive bees and crushed them into 500 μl of TEC to extract the SAP. This preparation of SAP proved less effective than synthetic IAA in triggering aggression (Fig. 2b; GLM, *P*=0.519 versus IAA but *P*=0.067 versus TEC), possibly because the final concentration of IAA was lower in the extract than in the solution prepared with synthetic IAA, or because not all the SAP components were soluble in TEC. Nevertheless, a significant reduction of aggression could also be observed on stimulation with Lol+SAP compared with stimulation with SAP alone (GLM, *P*=0.042). Although PhE also seemed to reduce aggression in the presence of SAP, the effect was not significant (GLM, *P*=0.152). However, SAP+PhE was the only mixture other than SAP+Lol that induced an aggression level significantly different from the one observed during IAA trials (GLM, *P*=0.040), thus confirming the clear blocking of aggression by Lol and Phe (Fig. 2a).

To determine whether these laboratory results can be transferred to the colony level, we conducted a field experiment in which we investigated whether Lol could also decrease aggressiveness in the more relevant context of nest defence. Bees at the hive entrance were exposed to an odour for 2 min and then confronted to a standard stimulation used to measure aggressiveness^29^,^30^ (a jiggling black leather flag) for 1 min, with the odour still present (Fig. 2c). Aggressiveness was measured as the number of stingers collected on the flag and the data were normalized per colony to correct for the different levels of overall aggressiveness displayed by the three colonies that participated in this experiment (see Methods for details). The average number of stingers collected did not differ between the trials in which bees were exposed to the solvent control TEC and the trials in which they were exposed to the control odour Lim (Fig. 2d, analysis of variance (ANOVA) with repeated measures, Bonferroni-corrected threshold *α*=0.016, *P*=1.000). However, when Lol was blown at the hive entrance, significantly fewer bees stung the leather flag (Fig. 2d, ANOVA with repeated measures, Bonferroni-corrected threshold *α*=0.016, *P*=0.014 versus TEC and *P*=0.014 versus Lim). Thus, the blocking of aggression by Lol observed in the laboratory assays could be reproduced in the test field at the colony level.

### IAA is not masked by floral compounds blocking aggression

When two odours are presented simultaneously to honeybees, one of these odours can potentially overshadow or block the other so that the bees only respond to the more salient odour^31^,^32^,^33^,^34^. This effect could explain the decrease in aggression induced by floral compounds such as PhE or Lol when presented with IAA. To examine this possibility, we conducted a series of experiments using the well-established olfactory conditioning of the proboscis extension reflex (PER)^35^,^36^ in which immobilized bees are trained with paired presentations of an odour (the conditioned stimulus or CS) and sucrose reward (the unconditioned stimulus or US). We conditioned bees with a single odour or odour mixture (absolute conditioning), to investigate whether IAA was masked by Lol and PhE, the effective plant odours blocking aggression.

**Figure 3:**
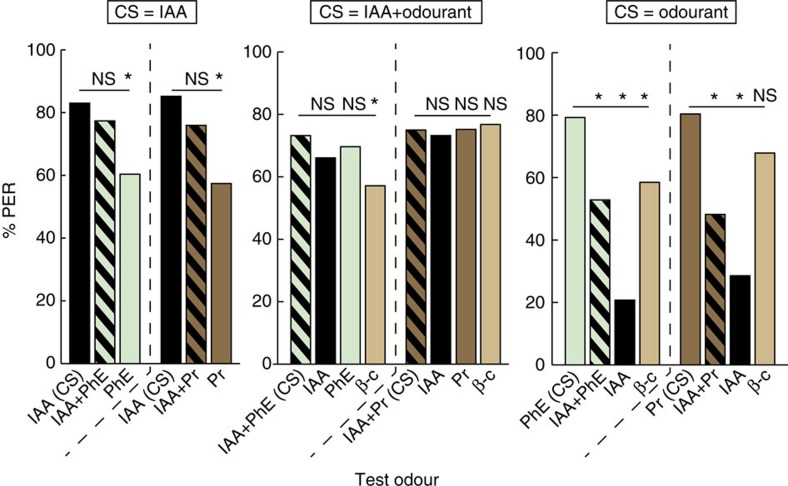
The impaired response to IAA was not caused by masking of this pheromone by the floral compounds. ‘CS=IAA': bees trained to associate a reward with IAA also responded well when IAA was mixed with the plant odour but less to the untrained plant odour alone. ‘CS=IAA+Odourant': bees trained with the mixture also responded to IAA and the plant odour when they were presented alone. However, in the case of Pr they also generalized to the novel plant odour β-c. ‘CS=Odourant': bees trained to the plant odour did not respond well when this odourant was mixed with IAA or when IAA alone was presented. Again, in the case of Pr they generalized to the novel plant odour β-c. McNemar tests, Bonferroni-corrected threshold *α*; NS, not significant; *P*>*α*, *P<*α*, *n*=53–56 bees per conditioned group.

In a first experiment, honeybees were trained to associate IAA (CS) with a sugar reward during four conditioning trials. Forty-five minutes after the end of the conditioning phase, bees were tested with the CS alone, the mixture of IAA+the plant odour and the plant odour alone. If IAA was masked by the plant odour in the mixture, the bees should respond significantly less to the mixture than to IAA. The plant odours used were PhE (*n*=53), as this molecule was effective in reducing the honeybee response to IAA, and Pr (*n*=54) as a control odour with no effect on aggression (see Fig. 2a). During the tests, honeybees trained to IAA responded similarly to IAA and to the mixtures containing IAA and a plant odour (Fig. 3, CS=IAA; McNemar tests, Bonferroni-corrected threshold *α*=0.025, *P*>*α* in both cases), but significantly less to the plant odour alone, which was novel to them (McNemar tests, Bonferroni-corrected threshold *α*=0.025, *P*<0.01 for both odourants). These results suggest that the mixtures were perceived by bees as being similar to IAA. However, these results do not allow us to conclude without doubt that the bees perceived IAA as a separate element of the mixture.

Therefore, in a second experiment, we conditioned groups of 56 honeybees with a mixture of IAA and one of the plant odours, Phe or Pr, as CS (that is, IAA+PhE or IAA+Pr). We then tested whether they would also respond to IAA alone, the plant odour alone or a novel odour β-caryophyllene (β-c). A high response to IAA would indicate that bees recognize IAA as one of the mixture components, thus precluding overshadowing by the plant odour. Indeed, honeybees responded similarly to IAA, the plant odour and the mixture (Fig. 3, CS=IAA+Odourant; McNemar tests, Bonferroni-corrected threshold *α*=0.016, *P*>*α* for all four odour conditions), thus suggesting that IAA and the plant odour are learnt and processed separately even if presented as a mixture during conditioning. After training to IAA+PhE, the response to the novel odour β-c was significantly lower than to the conditioned odour (McNemar test, Bonferroni-corrected threshold *α*=0.016, *P*=0.012). However, after training to IAA+Pr, the bees' response to β-c was high and similar to that of IAA+Pr (CS; Cochran *Q* test, *P*=0.914 on this data set as a whole). Thus, we cannot exclude that the bees' response to IAA was not due to a nonspecific response to all odourants (generalization), at least in the case of IAA+Pr training.

Hence, in a final experiment we trained the bees to one of the plant odours, Phe (*n*=53) or Pr (*n*=56), and quantified in subsequent tests their responses to the novel odourants IAA and β-c, and to the mixture of the plant odour conditioned and IAA, thus reversing the conditions of the first experiment (Fig. 3, CS=Odourant). The response to IAA, which was a novel odour in this case, was very low. Surprisingly, only few bees responded to the mixture that contained the conditioned plant odour. When compared with the percentage of bees responding to the plant odour on its own, these differences were highly significant (McNemar tests, Bonferroni-corrected threshold *α*=0.016, *P*<<*α* for these four test odours). Response to β-c as novel odour was also significantly lower for bees trained with PhE (McNemar test, Bonferroni-corrected threshold *α*=0.016, *P*=0.001) but not for bees trained with Pr (McNemar test, Bonferroni-corrected threshold *α*=0.016, *P*=0.039). This suggests that β-c may be perceptually similar to Pr for honeybees, so that bees trained with IAA+Pr respond to this odour on the basis of similarity rather than nonspecifically.

It is intriguing that few bees responded to the mixture of IAA+PhE or IAA+Pr after training with the plant odour alone (third experiment), whereas all learners responded to this same mixture after training with IAA (first experiment). This strongly suggests that IAA is the dominant component of the mixture, negatively affecting the perception of the plant odour. Indeed, IAA seems to mask the plant odour, which is not surprising considering that IAA is present at much higher concentrations in the mixture than the plant odour (10% versus 0.075%, Table 1). Bees conditioned to odour mixtures respond more to a dominant component in the mixture^32^,^33^. Based on this finding, bees trained to IAA+PhE or IAA+Pr should respond more to IAA and less to the plant odour given the concentration differences of these odourants. However, this was not the case (Fig. 3, CS=IAA+Odourant). This result can be due to the fact that appetitive conditioning to IAA induces high generalization levels to plant odours in honeybees^37^. Taken together, these experiments demonstrate that the decreased response to IAA observed during the aggression assay when some floral compounds were also present cannot be explained by a masking of IAA by these floral odours.

### Aggression-reducing odourants have an appetitive value

A possible explanation why certain floral compounds prevent bees from stinging in response to IAA could be that these compounds are associated with floral rewards and elicit feeding or foraging, thus preventing the bees from engaging into defence even in the presence of IAA. To test this hypothesis, we measured the spontaneous PER of honeybees participating in the colony defence (guards and soldiers collected as described above) when they were presented with the five floral odours (PhE, Lav, Lol, LiA and Lim) and with TEC as the solvent control (*n*=110). Each bee was presented with all six odours and the order of presentation was randomized between bees.

**Figure 4:**
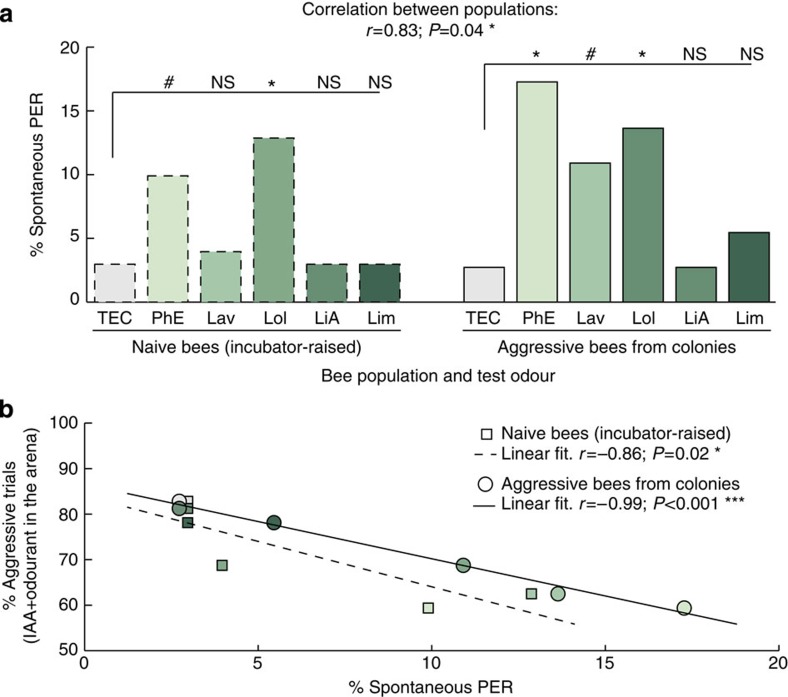
Floral compounds compete with IAA to an extent directly proportional to their appetitive value. (**a**) Bees participating in the colony defence or naive bees raised in an incubator exhibit spontaneous proboscis extension responses to some floral compounds, in particular to PhE and Lol. McNemar tests, Bonferroni-corrected threshold *α*=0.01; NS, not significant; *P*>*α*, #PhE *P*=0.020, #Lav *P*=0.013, *P<*α*, *n*=101 naive bees and *n*=110 aggressive bees. The responses of naive and aggressive bees are correlated. Pearson's *r* test, *r*=0.83; *P*=0.043. (**b**) The appetitive value of each floral compound correlates with the extent to which it affected the response to IAA during the aggression assays. Pearson's *r* tests, aggressive bees: *r*=−0.99, *P*<0.001; naive bees: *r*=−0.86, *P*=0.027.

Honeybees extended their proboscis significantly more often when exposed to PhE and Lol (McNemar test, Bonferroni corrected threshold α=0.01, *P*<α versus TEC for both odorants, Fig. 4a) but not when presented with LiA or Lim (McNemar test, Bonferroni-corrected threshold *α*=0.01, both *P*>*α* versus TEC), which had no effect in reducing aggression. As also observed during the aggression assay, Lav elicited an intermediate PER response, not as strong as Lol or PhE (McNemar test, Bonferroni-corrected threshold *α*=0.01, *P*=0.013 versus TEC; Fig. 4a). Further analysis of the spontaneous response data with regards to the aggression data (Fig. 2a) revealed a strong correlation between the appetitive value of the tested floral compounds and the extent to which they decreased recruitment by IAA (Pearson's *r* test, *r*=−0.99, *P*<0.001; Fig. 4b).

Spontaneous responses to floral odours are well known in honeybees and can be explained by prior foraging experience^38^. However, our experiments on aggression were conducted over a year using bee colonies that were free to forage all year round in a seasonally changing environment. Their experience with floral odours varied and, as a consequence, their olfactory processing and responses to plant odours should have varied accordingly^39^. How then can we explain the consistent effect over a whole year of Lol and PhE on aggression in our study? We postulated that the preference for certain floral odours found in the guards and soldiers that participated in our experiments, and whose main task is not foraging, may be already determined at the time of emergence rather than shaped by foraging experience. To test this hypothesis, we collected newly emerged bees from a brood frame and kept them in groups of 20 bees in cages in an incubator for 10 days with unscented sugar solution as food. After emergence, these bees were thus raised without any olfactory experience emanating from the colony, food stores or floral sources found in nature. After 10 days, we tested the spontaneous PER response of these ‘naive bees' to the same floral compounds (*n*=101). The naive bees exhibited a pattern of PER responses similar to that of the aggressive guard and soldier bees (Fig. 4a, Pearson's *r* test, *r*=0.83, *P*=0.043). In particular, we observed a higher level of responses to Lol than to the solvent TEC (McNemar test, Bonferroni-corrected threshold *α*=0.01, *P*=0.008), whereas PhE induced only marginally more proboscis extensions than TEC (McNemar test, Bonferroni-corrected threshold *α*=0.01, *P*=0.020). None of the other tested floral odours induced significant PER response in naive bees.

Crucially, the data set from the naive bees also correlates well with the results from the aggression assay (Pearson's *r* test, *r*=−0.86, *P*=0.027; Fig. 4b). This strongly suggests that the olfactory preferences of guard and soldier bees for the specific floral compounds Lol and PhE are already determined at the time of emergence, and that the appetitive value of Lol and PhE may be the factor that reduces honeybee aggression in the presence of IAA.

## Discussion

The aggressive behaviour of the honeybee is a considerable public health issue, with 0.3–7.5% of the population allergic to bee venom and a prevalence reaching 14–49% for beekeepers^40^. Understanding the biological mechanisms at play is a crucial step in developing tools for its management. Here we used a novel bioassay to investigate whether plant odours could decrease the aggressive behaviour of honeybees. We found that the floral compounds Lol and PhE reduce the aggressive response triggered by the alarm pheromone, thus exerting a calming effect on disturbed bees. We further show that this effect directly correlates with the appetitive value of the floral odours used as detractors from aggression: the higher the appetitive value, the lesser the aggression elicited by a concomitant exposure to alarm pheromone.

Our novel arena-based bioassay combines aggression-triggering elements detected by honeybees in nature and reliably induces bees to sting a target, while allowing the experimenter to easily record reproducible behavioural elements of aggression. This robust and technically simple assay has the potential to become a standard assay used to study the molecular and neural mechanisms underlying aggression in honeybees.

Using this assay, our first experiment challenged the view that single, individual honeybees rarely react to the alarm pheromone^15^. Our analysis shows that single bees do in fact react to IAA, but that in the absence of this pheromone the baseline aggressiveness of single bees is higher than for paired bees. Two alternative interpretations are possible for these results. Single bees may be more reactive to the dummy, possibly because being alone is a stressor in itself. Alternatively, paired bees could be less reactive, because being in a group would diminish the threat of the dummy (statistically). Importantly, previous studies reporting that single honeybees do not react to SAP determined the response to this pheromone as an increase in metabolic rate rather than as stinging behaviour^15^,^41^. This makes our first explanation more probable: if being alone is a stressor, then the metabolic rate of single bees would be high even before the presentation of SAP, rendering the detection of a metabolic change difficult.

Two types of pheromones are commonly distinguished: releaser pheromones, which provoke immediate and short-term responses, and primer pheromones, which cause long-term physiological changes, eventually leading to behavioural modifications. The honeybee SAP and its main component IAA belong to both categories. In addition to eliciting a defensive behaviour, IAA has long-lasting physiological effects on honeybees. First, it induces opioid-like analgesia, which is thought to prevent the bees from withdrawing from the fight^42^. Second, it impairs appetitive learning for up to 24 h after exposure^19^. In the latter case, it was concluded that IAA detracts the bees from responding to appetitive signals that are irrelevant in the context of colony defence. However, in our study we found that bees exposed to an appetitive floral odour exhibit reduced aggression levels. Although these results seem contradictory at first sight, a major difference between both studies is that our defensive encounter only lasted 3 min, whereas in the other study the bees experienced 30 min of IAA exposure before conditioning^19^. Thus, the learning impairment might reflect a slow behavioural shift that could play an important role during intense and long-lasting defensive events.

The crucial question remains why some floral odours (and not others) have an inhibiting effect on the response to the alarm pheromone. We demonstrated that these floral compounds were already appetitive to newly emerged honeybees, which were not in contact with combs or comb odourants since their emergence. This result supports the idea that some odourants may be innately appetitive to bees with no foraging experience. An alternative explanation could be, however, that the naive bees tested in our experiments were imprinted by these odourants during larval development if they were present in the wax comb. Nevertheless, it is unknown whether larval honeybees can learn olfactory cues and retain this information throughout the development and metamorphosis until the adult stage. On the contrary, innate preferences for colours are well known in flower-naive honey bees^43^, which correlate with the colour of flowers producing high-quality nectar rewards^43^. In the same manner, innate preparedness for floral odour cues could help inexperienced bee to find food sources in their first foraging flights^44^. Interestingly, flower-naive honey bees do not land on artificial coloured flowers unless they are scented^43^.

Both Lol and PhE, but none of the other compounds we tested, have previously been shown to elicit spontaneous appetitive responses in honeybees^45^. Similarly, these two compounds often feature among the key components that the bees use to learn complex mixtures^32^,^46^. Nonetheless, it is, to our knowledge, the first time that the existence of preferences for some floral odours has been formally shown using naive honeybees whose exposure to these odours during adult life has been controlled for. Further work would be needed to determine whether the slight differences observed between the preferences of naive bees and guard/soldier bees are caused by a refinement through olfactory experience or by further maturation of the olfactory system after 10 days.

The most striking result, however, is not the existence of olfactory preferences but the fact that exactly the odours that are associated with reward are the ones that affect IAA-triggered aggression. After having excluded perceptual interference during olfactory processing of plant odours and IAA via the PER assay, this is the first lead towards the underlying regulatory mechanisms how Lol and PhE may block aggressive behaviour. The fact that exposure to IAA reduces learning of floral odours in an appetitive context^19^, and that floral odours reduce in turn the response to IAA in an aggression context implies that an integrative mechanism in the bee brain has to weigh different odour values, in different contexts, against each other.

**Figure 5:**
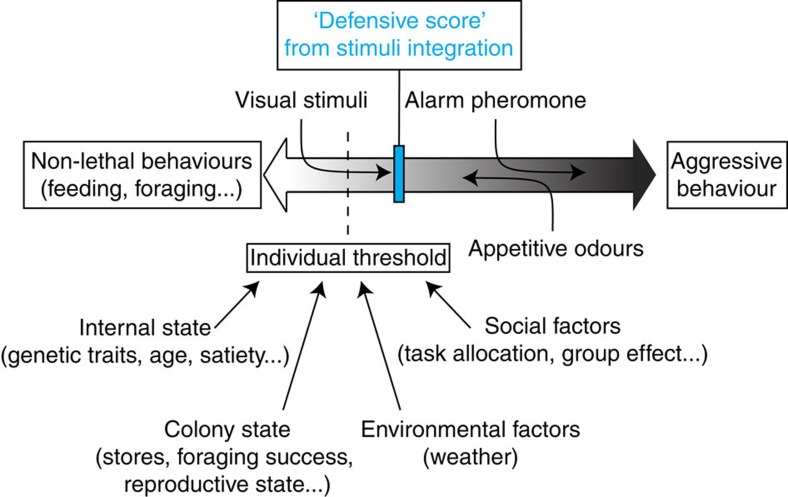
A possible model for the decision-making process underlying honeybee aggression. We postulate the existence of an integrative mechanism, which computes a ‘defensive score' from all the stimuli (visual, mechanical, olfactory and so on). This score, represented here as the blue cursor, is compared against an individual threshold determined by internal and colony state, as well as by environmental and social factors. Appetitive floral odours and the alarm pheromone exert opposite actions on the defensive score moving it towards and away from the individual threshold, respectively. The resulting state determines whether the bee engages into active defence.

Numerous studies support the idea that the division of labour in honeybee colonies is caused by differences in response thresholds to environmental stimuli^7^,^47^,^48^,^49^. Based on this model and on our new findings, we present a possible mechanism for the decision-making process underlying honeybee aggression (Fig. 5). In this model, we postulate the existence of an integrative mechanism in the bee brain, which weighs the different stimuli (olfactory but also visual and mechanical) and computes an overall ‘defensive score'. This score would then be compared with an individual threshold, to choose between possible behavioural outputs, which in our model are limited to engaging into defence or continuing to perform other non-lethal colony duties (for example, foraging). The individual threshold itself would be determined by a range of factors including the internal state of the bee^16^,^50^ and the state of the colony^11^,^12^,^16^,^51^. We also suggest that this individual threshold might already take into account social^14^,^52^,^53^ and environmental^54^ factors as a way of enhancing the computational speed of the integrative mechanisms, but these parameters may also be considered as changing stimuli and feed directly into the integrative mechanism. The changing individual threshold along with variations in the weight attributed to each stimulus would thereby create the diversity of reactions observed during a defensive event. Our findings constitute a first step towards the elucidation of the mechanisms regulating honeybee aggression. Further research may also shed light on the adaptive evolutionary value of plant odours modulating this complex and little understood behaviour. For example, floral odours are usually encountered during foraging trips away from the colony, a context in which stinging is not a primary adaptive response. Therefore, floral odours may detract bees from aggressive interactions by acting as markers of distant foraging locations. A decrease in aggressiveness correlated with the perceived distance from the nest has already been demonstrated in another social insect, the desert ant^55^. In the honeybee, a similar effect could be triggered by the perception of appetitive floral odours.

## Methods

### Honeybees

For all experiments, except the field test, bees were collected from several unrelated honeybee colonies (*A. mellifera ligustica*) housed on the University of Queensland St. Lucia campus (Brisbane, Queensland, Australia), from April 2013 to October 2014, excluding the winter months—June to August. All colonies were freely foraging and underwent routine beekeeping inspections and honey collection during the course of the experiments. An equal number of bees from four different colonies participated. The bees were caught on sunny days in two rounds (around 0930 and 1100, h, alternatively for each colony), in a pattern ensuring that no colony was disturbed more than once every 48 h. This delay allowed the hives to fully settle down in between disturbances and indeed no increase in aggressiveness was observed over time. To select for the population of bees involved in colony defence (guards and soldiers), the bees were collected by waving a large black feather in front of the hive entrance for a few seconds. Once the feather was covered in about 30–40 attacking bees, it was quickly placed into a sealable plastic bag and in a freezer at −20 °C. The state of the bees was checked after 5 min and then every 1–2 min until they were all motionless (on average 8.25 min in the freezer). The bees showing the quickest recovery were selected and placed alone or in pairs into 50 ml syringes (Terumo) containing a wet tissue and three droplets of sugar water (50% sugar water, vol/vol). The tip of the syringe was cut and replaced with a plastic sliding door held with a paper clip. In case of pairs, one honeybee was marked with a red dot on the thorax (enamel paint), while the other was left unmarked. Similarly, half of the single honeybees were marked in the same manner, while the other half remained unmarked, to control for a possible effect of the enamel paint. The data revealed no difference in aggressive behaviour between marked and unmarked bees (*χ*^2^=1.575, df=2, *P*=0.455). Once this step was complete, all honeybees were allowed to recover for another 10 min and up to 80 min before being tested in the set-up investigating aggressive behaviour. If one or both bees showed signs of poor recovery when put in the set-up (difficulty to hold upside down, clumsy and/or slow walk), the whole trial was excluded from further analysis. All the materials used to contain the bees were washed with detergent, rinsed and dried after each use.

For the main aggression experiment, a large number of different odourants were tested. As it was not technically possible to test them all simultaneously, the odourants were distributed into four sets, each including IAA and the solvent TEC as reference points. As there was no statistical difference between these references across the four sets (see results), the data were pooled. As a consequence, the IAA and TEC groups include 128 pairs of bees, whereas every other group includes 32 pairs. The experiment testing the role of social interactions included 32 pairs or individuals per group. This sample size was chosen based on pilot experiments. The experiment using the full SAP included 48 pairs of bees per odour condition. The sample size for this experiment was increased, to gain the statistical power necessary to detect this smaller effect. No bee was tested more than once nor released (they were killed).

The field test was performed at the apiary of the University Paul Sabatier (Toulouse, France) at the end of summer 2015 (August–September). Three colonies of the same subspecies (*A. mellifera ligustica*) participated in this experiment. They were all freely foraging, treated against Varroa and underwent routine beekeeping inspections during the course of the experiment. The colonies were tested no more than once every 24 h, in the morning of sunny days.

### Odourants

All odourants were pure chemicals (98–99.9% purity) from Sigma-Aldrich and kept in the freezer (−20 ° C). Before each set of experiments, a fresh batch of odourant dilutions was prepared using TEC for solvent and kept for the whole length of this experiment. These odourants were delivered at room temperature (25 ° C) and kept in the fridge (4 ° C) when not in use. Table 1 presents all the odourants used and their concentrations. The concentration of 0.075% (vol/vol) for all plant odours was chosen taking as reference the concentration of Pr, the combination of plant-derived odours used in our work (0.03% Z-3-hexen-1-ol, 0.03% E-2-hexenal and 0.015% α-pinene in TEC). This concentration was shown to reduce corticosterone, glucose and redox responses elicited by psychological stress in rats^25^,^56^, and was thus used as a starting point for our study. For the field test, the concentration of the odourants was increased to 1%, to cope with the large volume of air in which the odour had to be delivered.

### Aggression assay

Assessment of the bees' aggressiveness was done in circular arenas (Fig. 1a, 14 cm diameter, 4 cm high) made of transparent plastic. A sliding door on the side allowed introduction of the honeybees from the syringes. The various odourants used were blown into the arena through three entry points (4 mm ID) regularly spaced and at middle height along the wall. The arena lid was regularly drilled with about 40 holes (1 mm ID), to avoid building up of the odour inside the arena. A 1-cm hole was also opened in the middle of the arena floor to allow passage of the step motor axle (Aviosys DYO AK27PCB). The step motor was connected to a DC power unit set to 9 V and 0.25 A. Before each trial, the arena was wiped clean using a 70% ethanol solution and a wet filter paper was put on the floor to maintain the humidity. A dummy was placed horizontally on top of the step motor axle with blue tack. Four dummies were made, each consisting of the barrel of a 3-ml syringe (cylinder of 5–6 cm long, 1 cm in diameter) covered with a rectangular patch of black suede leather (4.5 × 7 cm) and prolonged on one end with a soft black feather. The use of the four dummies was always balanced across the different conditions. The leather patch was held with four pieces of yellow electrical tape and was changed whenever it had been stung. Stung leather patches were rinsed with clear water and left to dry outside for at least 24 h before being used again; the feather was also cleaned with 70% ethanol. To increase the jerkiness of the movement, the step motor was used at its lowest speed: as a result, the dummy rotated horizontally across the middle of the arena floor, while the black feather gently brushed the sides. The size and shape of the dummy allowed the honeybees to freely move along the sides and lid of the box without touching it. The purpose of the black feather was to disturb the bees without causing them pain. Indeed, this feather was merely touching the bees and was not strong enough to change the path of a walking honeybee.

### Odour delivery in the arena

Medical grade air (BOC, North Ryde, Australia) was delivered from a 680-l tank and fed into a custom-designed olfactory stimulus controller. This olfactometer delivered a constant clean air flow of 1 l min^−1^. Polytetrafluoroethylene (PTFE) Teflon tubing (3 mm ID) led this air flow to the base of a 15-ml Falcon tube, inside which filter papers carrying the odourants were placed. Further up the sides of the Falcon, three more Teflon pipes were connected, which terminated on the other end into truncated pipette tips. The resulting device could be easily plugged in and out of the three odourant entry points of the arena (Fig. 1). To avoid contamination, eight of these devices were made and each one was used for the delivery of a single odourant (or combination of odourants) during the course of an experiment. In between experiments, they were thoroughly washed with 70% ethanol and left to dry for at least 24 h before being used for another set of odours.

During each trial, two pieces of filter papers were put in the Falcon tube dedicated to the odour delivery. Depending on the odour combination tested, they were either both blank (none, no odour control), one soaked with 10 μl of an odourant and the other with 10 μl of solvent (odourant alone) or one soaked with 10 μl of an odourant and the other with 10 μl of the alarm pheromone (IAA+odourant or SAP+odourant). For example, for the TEC control both papers were soaked with TEC, for testing Lim alone the combination was Lim+TEC and for testing the interaction between Lim and the alarm pheromone one filter paper carried IAA and the other Lim. To ensure homogeneity of the data, presentation of the different odourants was balanced over colonies and time of the day.

### Trials and scoring of the aggression assay

All trials were recorded with an high definition camera positioned above the arena. Each trial went as follows: first, the camera and the step motor moving the dummy were switched on. The tip of the syringe containing the honeybees was then inserted inside the arena. The olfactometer was always switched on just before introduction of the honeybees in the arena, while the arena door was already open but not yet the syringe door. As a result, the bees received a quick puff of odour just before facing the dummy, thus mimicking the successive steps of colony defence usually occurring in nature. The syringe door was then opened and, if necessary, the bees were gently pushed inside the arena with the plunger. The odourant air flow was left running during the whole length of a trial (3 min). During the trial, the rotating direction of the dummy was manually and randomly changed multiple times.

The stinging response of a bee was scored visually and defined as the bee holding onto the dummy for at least 3 s, with the tip of the abdomen pressed against it in the characteristic stinging position; the vast majority (90.2%) of the attacks recorded were further confirmed by the presence of the stinger apparatus still embedded in the dummy leather. Another 5.7% of the aggressive bees stayed exclusively on the feather, which was considered the reason why their stinger was not pulled away. Finally, the remaining 4.1% of attacks scored correspond to bees either choosing to bite the dummy, or (in very few cases) to bees clearly attempting to sting the dummy, although the reason why the stinger could not be recovered was unknown. Fewer than 1% of the trials were considered borderline (for example, when an agitated bee contacted the dummy multiple times but did not exhibit any of the other criteria) and were excluded. For each trial, the aggressive response was scored as 1 if at least one of the bees attacked the dummy and 0 if all bees remained calm.

### Field test

Before the beginning of the experiment, the size of the landing board was standardized (5.5 × 53 cm) for all the colonies and an open box was created around the hive entrance by placing two vertical wooden walls (10 cm high) on each end of the landing board and a transparent plastic roof on top (Fig. 2c). This box was closed at the beginning of each test by the addition of a front door, thus creating a stable atmosphere of about 2.5 l at the hive entrance in which an odour could be delivered. To this end, two 15 ml Falcon tubes containing a filter paper carrying 10 μl of the odour were inserted into holes in the lateral walls (Fig. 2c). Four small holes were drilled at the bottom and the lid was modified so that the tubes could be easily connected to the output of an aquarium air pump (Rena 300, delivering a total air flow of about 3.3 l min^−1^). To avoid contaminations, a pair of tubes was made for each odour tested. To measure aggressiveness at the colony level, a black leather patch (4.5 × 7 cm) on a wooden pole was placed in front of the hive entrance, 1–2 cm away from the landing board, and jiggled via a small motor^29^,^30^ (Lego Power Functions XL-Motor). A square marker on the ground ensured that the flag positioning was the same across days. The tests started by closing the front door of the box and switching on the air pump to deliver the odour, while the flag remained motionless just outside the box. The order of presentation of the odourants was randomized. After 2 min of odourant exposure, the flag motor was switched on and the door was removed, thus allowing the bees to confront the moving flag (with the odour delivery still on; Fig. 2c). This step lasted for another minute (hence a total of 3 min for the whole test), after which the motor was stopped and the flag quickly sealed in a plastic box so that no additional bees could access it. The number of stingers embedded in the leather was then counted and used as a measure for aggressiveness at the colony level. The flags were discarded after they were stung and all the material was washed with 70% ethanol between trials. All trials were recorded with a camera placed above the landing board. Each of the three colonies was tested six times with each odour (*n*=18 per group) and data were normalized per colony (see below) to account for different inter-colony aggressiveness before being pooled.

### Masking experiment

In the morning, equal numbers of bees from the four colonies were caught at the hive entrance, using Falcon tubes. They were then cold anaesthetized in the freezer during 5 min and tethered in the restraining tubes used for PER conditioning^57^. They were fed with a droplet of sugar water (50% vol/vol) before being placed in a dark incubator (26 °C, 85% humidity) for 3 h. This is a standard procedure to homogenize the satiation level of the bees and habituate them to the restraining tube^57^.

The conditioning of the PER is a classical conditioning assay in which harnessed bees learn to associate odourants with the appetitive reward of sucrose solution^36^. When the antennae of a hungry, harnessed bee are touched with sucrose solution, the animal reflexively extends its proboscis to reach out to and suck the sucrose (PER). If an odourant is presented immediately before sucrose solution (forward pairing), an association is formed, which enables the odourant to release the PER in a following test. In our experiments, bees were exposed to an odour (CS) for 6 s followed by the presentation of sucrose solution (US, 50% vol/vol) for 3 s. The CS and US overlapped during 3 s. Bees were conditioned with four trials spaced by 13 min. Forty-five minutes after the last conditioning trial, the bees' responses to three or four odours was tested, in a randomized order and without any sugar reward. There was a 13 min inter-trial interval between the tests. Three sets of experiments were conducted. In the first set, the bees were conditioned with IAA and tested either with IAA, PhE and IAA+PhE or with IAA, Pr and IAA+Pr. In a second set, the bees were trained with a mixture (IAA+PhE or IAA+Pr) and tested with the same mixture, IAA, the plant odour alone (PhE or Pr) and β-c (novel odour). Finally, in a third set of experiments the bees were trained with the plant odour (PhE or Pr) and tested with the same plant odour, the corresponding mixture (IAA+PhE or IAA+Pr), IAA and β-c. These six test groups include respectively 53, 54, 56, 56, 53 and 56 honeybees. These sample sizes are within the standard range used to ensure statistical power during analysis of PER experiments^57^.

### Experiment testing the appetitive value of odours

Two populations of honeybees were tested during this experiment. Defensive bees were caught directly from colonies as described above. To test whether some odours were innately appetitive, we produced odour-naive bees by placing a capped brood frame in a dark incubator (34 °C) and collecting the newly emerged bees every day. Groups of 20 age-matched bees were then raised in meshed cages in the same incubator for 10 days. They had *ad libitum* access to water and an unscented sugar solution (50% vol/vol), except during the night before testing when the sugar solution was removed to increase their motivation. Fresh food and water were provided every day. All the bees were cold anaesthetized on the morning of the test day, placed in the restraining tubes used for PER testing, fed a droplet of sugar water and then left in a 26 °C dark incubator for 4 h before testing. A total of 101 naive bees and 110 aggressive bees participated in this experiment.

Each bee was presented once with the six odours tested, in a randomized order and spaced by 13 min. Importantly, no training was performed before testing and no reward was given during testing. At the end of the testing session, the PER was triggered by touching the honeybees' antennae with sugar water and the few bees that did not respond to this stimulation were excluded from the analysis.

### Statistics and calculation of theoretical data

We used *χ*^2^-tests to analyse the data produced by the experiment investigating the role of social interactions as the observations were independent and all expected cell counts were >10. To calculate the theoretical data, we considered that the frequency of aggressive trials for single bees under given conditions represent the probability *p* of one bee from this population to sting under these conditions. The probability of scoring an aggressive trial from two such bees was then calculated using the classical probability laws for two independent events. As a result,


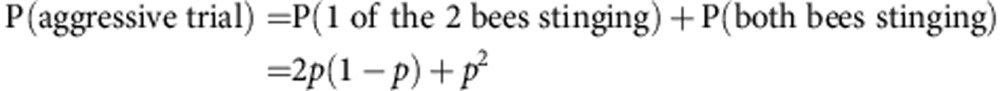


Or more generally,


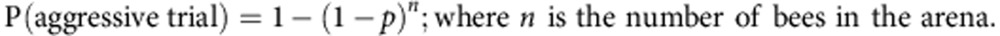


The expected results were then obtained by multiplying this probability by the sample size.

All the other aggression data were analysed using a GLM set-up with a logit link function appropriate for binomial data.

In the field test data set, two outliers had to be removed in each group. They all corresponded to extremely aggressive trials during which two to nine times more stingers than usual were collected. Removing them did not change the overall pattern of responses observed but allowed the data set to meet the normality assumption (Shapiro–Wilk tests) necessary to run an ANOVA with repeated measures. The data were also normalized per colony by subtracting the colony average from each data point and dividing by the colony s.d. (standard score). This was done to homogenize the data, as each colony had a different baseline aggression level (from 1.76 to 17.6 responding bees on average for the most aggressive colony). *Post hoc* pairwise comparisons were corrected with a Bonferroni procedure.

A potential difference between the percentages of bees exhibiting a PER response when presented with the different odourants was tested with Cochran *Q* test, as it is adapted to repeated measures with dichotomous responses. If this test was significant, a *post hoc* analysis was performed using multiple McNemar tests and a significance threshold adjusted with a Bonferroni correction. The correlation between two data sets was tested using Pearson's *r* test.

## Additional information

**How to cite this article:** Nouvian, M. *et al.* Appetitive floral odours prevent aggression in honeybees. *Nat. Commun.* 6:10247 doi: 10.1038/ncomms10247 (2015).

## Figures and Tables

**Table 1:** Chemical and biological information on the odourants used to investigate the effect of olfactory cues on honeybee aggression.

**Odourant**	**Abbreviation**	**Composition**	**Background information**	
None	None	NA	Control: no odour.	Controls
Triethyl citrate	TEC	pure	Control: solvent. Odourless.	
2-Phenylethanol	PhE	0.075% 2-PhE in TEC	Common floral compound	
Lavender	Lav	0.04% LiA+0.035% Lol in TEC	Lavender odour simplified to its two main components	
Linalool	Lol	0.075% Lol in TEC	Common floral compound (inc. lavender)	Floral odours
Linalyl acetate	LiA	0.075% LiA in TEC	Common floral compound (inc. lavender)	
R-(+)-Limonene	Lim	0.075% Lim in TEC	Common floral compound	
Praescent	Pr	0.03% *Cis*-3-hexanol+0.03% *trans*-2-hexenal+0.015% α-pinene in TEC	Green odour. Decreases the noxious effects of chronic stress on mice and humans^25^,^26^,^56^	Plant odours
β-Caryophyllene	β-c	0.075% β-c in TEC	Found in many essentials oils (e.g., clove, rosemary)	
Citral	Ci	0.075% Ci in TEC	Main component of the Nasanov pheromone, attractant	
Iso-amyl acetate	IAA	10% IAA in TEC	Main component of the SAP	Pheromones
Sting alarm pheromone	SAP	30 Stings crushed in 500 μl TEC	Complete alarm pheromone extracted from the sting	

NA, not applicable.

## References

[b1] Moore A. J., Breed M. D. & Moor M. J. The guard honey-bee—ontogeny and behavioral variability of workers performing a specialized task. Anim. Behav. 35, 1159–1167 (1987).

[b2] Free J. B. The stimuli releasing the stinging response of honeybees. Anim. Behav. 9, 193–196 (1961).

[b3] Breed M. D., Robinson G. E. & Page R. E. Division of labor during honey bee colony defense. Behav. Ecol. Sociobiol. 27, 395–401 (1990).

[b4] Maschwitz U. Alarms substances and alarm behaviour in social hymenoptera. Nature 204, 323–327 (1964).

[b5] Boch R., Shearer D. A. & Stone B. C. Identification of isoamyl acetate as an active component in the sting pheromone of the honey bee. Nature 195, 1018–1020 (1962). 13870346 10.1038/1951018b0

[b6] Collins A. M. & Blum M. S. Alarm responses caused by newly identified compounds derived from the honeybee sting. J. Chem. Ecol. 9, 57–65 (1983). 24408619 10.1007/BF00987770

[b7] Collins A. M., Rinderer T. E., Tucker K. W., Sylvester H. A. & Lackett J. J. A model of honeybee defensive behavior. J. Apic. Res. 19, 224–231 (1980).

[b8] Haydak M. H. How long does a bee live after losing sting. Glean. Bee Cult. 79, 85–86 (1951).

[b9] Hermann H. R. Sting autotomy, a defensive mechanism in certain social hymenoptera. Insectes Soc. 2, 111–120 (1971).

[b10] Shorter J. R. & Rueppell O. A review on self-destructive defense behaviors in social insects. Insectes Soc. 59, 1–10 (2012).

[b11] Ribbands C. R. The defence of the honeybee community. Proc. R. Soc. Lond. Ser. B Biol. Sci. 142, 514–524 (1954). 13215508 10.1098/rspb.1954.0040

[b12] Collins A. M. & Rinderer T. E. Effect of empty comb on defensive behavior of honeybees. J. Chem. Ecol. 11, 333–338 (1985). 24309964 10.1007/BF01411419

[b13] Breed M. D. & Rogers K. B. The behavioral genetics of colony defense in honeybees: genetic variability for guarding behavior. Behav. Genet. 21, 295–303 (1991). 1863261 10.1007/BF01065821

[b14] Hunt G. J., Guzmán-Novoa E., Uribe-Rubio J. L. & Prieto-Merlos D. Genotype-environment interactions in honeybee guarding behaviour. Anim. Behav. 66, 459–467 (2003).

[b15] Moritz R. F. A. & Burgin H. Group response to alarm pheromones in social wasp and the honeybee. Ethology 76, 15–26 (1987).

[b16] Paxton R. J., Sakamoto C. H. & Rugiga F. C. N. Modification of honey bee (*Apis mellifera* L.) stinging behavior by within-colony environment and age. J. Apic. Res 33, 75–82 (1994).

[b17] Sandoz J. C. Behavioral and neurophysiological study of olfactory perception and learning in honeybees. Front. Syst. Neurosci. 5, 98 (2011). 22163215 10.3389/fnsys.2011.00098 PMC3233682

[b18] Sandoz J. C., Deisig N., de Brito Sanchez M. G. & Giurfa M. Understanding the logics of pheromone processing in the honeybee brain: from labeled-lines to across-fiber patterns. Front. Behav. Neurosci. 1, 5 (2007). 18958187 10.3389/neuro.08.005.2007 PMC2525855

[b19] Urlacher E., Frances B., Giurfa M. & Devaud J. M. An alarm pheromone modulates appetitive olfactory learning in the honeybee (*Apis mellifera*). Front. Behav. Neurosci. 4, 157 (2010). 20838475 10.3389/fnbeh.2010.00157 PMC2936933

[b20] Chaffiol A. *et al.* Pheromone modulates plant odor responses in the antennal lobe of a moth. Chem. Senses 39, 451–463 (2014). 24798893 10.1093/chemse/bju017

[b21] Chaffiol A. *et al.* Plant odour stimuli reshape pheromonal representation in neurons of the antennal lobe macroglomerular complex of a male moth. J. Exp. Biol. 215, 1670–1680 (2012). 22539734 10.1242/jeb.066662

[b22] Namiki S., Iwabuchi S. & Kanzaki R. Representation of a mixture of pheromone and host plant odor by antennal lobe projection neurons of the silkmoth *Bombyx mori*. J. Comp. Physiol. A 194, 501–515 (2008). 10.1007/s00359-008-0325-3 18389256

[b23] Party V., Hanot C., Said I., Rochat D. & Renou M. Plant terpenes affect intensity and temporal parameters of pheromone detection in a moth. Chem. Senses 34, 763–774 (2009). 19770215 10.1093/chemse/bjp060

[b24] Reddy G. V. & Guerrero A. Interactions of insect pheromones and plant semiochemicals. Trends Plant Sci. 9, 253–261 (2004). 15130551 10.1016/j.tplants.2004.03.009

[b25] Spiers J. G., Chen H. J., Sernia C. & Lavidis N. A. A combination of plant-derived odors reduces corticosterone and oxidative indicators of stress. Chem. Senses 39, 563–569 (2014). 24935864 10.1093/chemse/bju026

[b26] Spiers J. G., Chen H. J. & Lavidis N. A. Stress alleviating plant-derived ‘green odors': behavioral, neurochemical and neuroendocrine perspectives in laboratory animals. Phytochem. Rev. 14, 713–725 (2015).

[b27] Butler C. G. & Calam D. H. Pheromones of the honey bee—the secretion of the nassanoff gland of the worker. J. Insect Physiol. 15, 237–244 (1969).

[b28] Collins A. M. & Blum S. M. Bioassay of compounds derived from the honeybee sting. J. Chem. Ecol. 8, 463–469 (1982). 24414957 10.1007/BF00987794

[b29] Collins A. M. & Kubasek K. J. Field-test of honey bee (Hymenoptera, Apidae) colony defensive behavior. Ann. Entomol. Soc. Am. 75, 383–387 (1982).

[b30] Guzman-Novoa E., Hunt G. J., Uribe J. L., Smith C. & Arechavaleta-Velasco M. Confirmation of QTL effects and evidence of genetic dominance of honeybee defensive behavior: results of colony and individual behavioral assays. Behav. Genet. 52, 95–102 (2002). 12036115 10.1023/a:1015245605670

[b31] Guerrieri F., Lachnit H., Gerber B. & Giurfa M. Olfactory blocking and odorant similarity in the honeybee. Learn. Mem. 12, 86–95 (2005). 15805307 10.1101/lm.79305 PMC1074325

[b32] Reinhard J., Sinclair M., Srinivasan M. V. & Claudianos C. Honeybees learn odour mixtures via a selection of key odorants. PLoS ONE 5, e9110 (2010). 20161714 10.1371/journal.pone.0009110 PMC2817008

[b33] Schubert M., Sandoz J. C., Galizia G. & Giurfa M. Odourant dominance in olfactory mixture processing: what makes a strong odourant? Proc. R. Soc. Lond. Ser. B: Biol. Sci. 282, pii: 20142562 (2015). 10.1098/rspb.2014.2562 PMC4344151 25652840

[b34] Smith B. H. Analysis of interaction in binary odorant mixtures. Physiol. Behav. 65, 397–407 (1998). 9877404 10.1016/s0031-9384(98)00142-5

[b35] Bitterman M. E., Menzel R., Fietz A. & Schafer S. Classical conditioning of proboscis extension in honeybees (*Apis mellifera*). J. Comp. Psychol. 97, 107–119 (1983). 6872507

[b36] Giurfa M. & Sandoz J. C. Invertebrate learning and memory: Fifty years of olfactory conditioning of the proboscis extension response in honeybees. Learn. Mem. 19, 54–66 (2012). 22251890 10.1101/lm.024711.111

[b37] Sandoz J. C., Pham-Delegue M. H., Renou M. & Wadhams L. J. Asymmetrical generalisation between pheromonal and floral odours in appetitive olfactory conditioning of the honey bee (*Apis mellifera* L.). J. Comp. Physiol. A. Neuroethol. Sens. Neural. Behav. Physiol. 187, 559–568 (2001). 10.1007/s003590100228 11730303

[b38] Gerber B. *et al.* Honey bees transfer olfactory memories established during flower visits to a proboscis extension paradigm in the laboratory. Anim. Behav. 52, 1079–1085 (1996).

[b39] Claudianos C. *et al.* Odor memories regulate olfactory receptor expression in the sensory periphery. Eur. J. Neurosci. 39, 1642–1654 (2014). 24628891 10.1111/ejn.12539

[b40] Bilo B. M. *et al.* Diagnosis of Hymenoptera venom allergy. Allergy 60, 1339–1349 (2005). 16197464 10.1111/j.1398-9995.2005.00963.x

[b41] Moritz R. F. A., Southwick E. E. & Breh M. A metabolic test for the quantitative analysis of alarm behavior of honeybees (*Apis mellifera* L.). J. Exp. Zool. 235, 1–5 (1985).

[b42] Núñez J., Almeida L., Balderrama N. & Giurfa M. Alarm pheromone induces stress analgesia via an opioid system in the honeybee. Physiol. Behav. 63, 75–80 (1998). 10.1016/s0031-9384(97)00391-0 9402618

[b43] Giurfa M., Núñez J., Chittka L. & Menzel R. Color preferences of flower-naive honeybees. J. Comp. Physiol. A 177, 247–259 (1995).

[b44] Butler C. G. The importance of perfume in the discovery of food by the worker honeybee (*Apis mellifera* L.). Proc. R. Soc. Lond. Ser. B Biol. Sci. 138, 403–413 (1951).

[b45] Dötterl S. & Vereecken N. J. The chemical ecology and evolution of bee-flower interactions: a review and perspectives. Can. J. Zool. 88, 668–697 (2010).

[b46] Pham-Delegue M. H. *et al.* Behavioural discrimination of oilseed rape volatiles by the honeybee *Apis mellifera* L. Chem. Senses 18, 483–494 (1993). 10.1093/chemse/22.4.391 9279462

[b47] Robinson G. E. Modulation of alarm pheromone perception in the honey bee: evidence for division of labor based on hormonally regulated response thresholds. J. Comp. Physiol. A 160, 613–619 (1987).

[b48] Pankiw T. in Swarm Intelligence Symp. 2005. SIS 2005. Proc. 2005 IEEE 1–6IEEE (2005).

[b49] Page R. E. Jr. & Amdam G. V. The making of a social insect: developmental architectures of social design. Bioessays 29, 334–343 (2007). 17373656 10.1002/bies.20549 PMC2398704

[b50] Breed M. D., Guzman-Novoa E. & Hunt G. J. Defensive behavior of honey bees: organization, genetics, and comparisons with other bees. Annu. Rev. Entomol. 49, 271–298 (2004). 14651465 10.1146/annurev.ento.49.061802.123155

[b51] Delaplane K. S. & Harbo J. R. Effect of queenlessness on worker survival, honey gain and defence behaviour in honeybees. J. Apic. Res. 26, 37–42 (1987).

[b52] Giray T. *et al.* Genetic variation in worker temporal polyethism and colony defensiveness in the honey bee, *Apis mellifera*. Behav. Ecol. 11, 44–55 (2000).

[b53] Moritz R. F. A., Southwick E. E. & Harbo J. B. Genetic analysis of defensive behavior of honeybee colonies (*Apis mellifera* L.) in a field test. Apidologie 18, 27–41 (1987).

[b54] Southwick E. E. & Moritz R. F. A. Effect of meteorological conditions on defensive behaviour of honey bees. Int. J. Biometeor. 31, 259–265 (1987).

[b55] Knaden M. & Wehner R. Path integration in desert ants controls aggressiveness. Science 305, 60 (2004). 15232099 10.1126/science.1097165

[b56] Einstein R. & Lavidis N. A. Methods of relieving stress. Patent WO2007022589 A1 (2007).

[b57] Matsumoto Y., Menzel R., Sandoz J. C. & Giurfa M. Revisiting olfactory classical conditioning of the proboscis extension response in honey bees: a step toward standardized procedures. J. Neurosci. Methods 211, 159–167 (2012). 22960052 10.1016/j.jneumeth.2012.08.018

